# Supplementary material for: Blocked transcription-translation complexes are rescued by transcript release followed by trans-translation
Source: J Bacteriol. 2026 Aug 6;208(9):e00270-26. doi: 10.1128/jb.00270-26 (PMC13584721; doi:10.1128/jb.00270-26)
Supplement: Supplementary file 1 [file jb.00270-26-s0001.pdf]

Table S1. List of peptides identified by LC-MS/MS.

Peptide sequence	Sequest Xcorr[a]
DDGNYKTRAEVKFEG	2.53
DFFKSAMPEGYVQERTIFFK	2.74
DFFKSAMPEGYVQERTIFFKD	3.12
DFFKSAMPEGYVQERTIFFKDDGNYKTRAEVKFEG	1.67
DGNILGHKLEYNYNSHNVYIMA	3.77
DGNYKTRAEVKFEG	2.62
DGPVLLPDNHYLSTQSALSK	3.89
DGPVLLPDNHYLSTQSALSKDPNEKR	3.7
DHYQQNTPIGDGPVLLP	4.73
DHYQQNTPIGDGPVLLPDNHYLSTQSALSK	4.68
DKQKNGIKVNFKIRHNIE	2.04
DNHYLSTQSALSK	3.55
DNHYLSTQSALSKDPNEKR	3.72
DTLVNRIELKGIDFKE	3.72
DTLVNRIELKGIDFKEDGNILGHKLEYNYNSHNVYIMA	1.23
DDGNYKTRAEVKFEG	2.53
DFFKSAMPEGYVQERTIFFK	2.74
DFFKSAMPEGYVQERTIFFKD	3.12
DFFKSAMPEGYVQERTIFFKDDGNYKTRAEVKFEG	1.67
DGNILGHKLEYNYNSHNVYIMA	3.77
DGNYKTRAEVKFEG	2.62
DKQKNGIAANDHHHHHHD	1.72
DTLVNRIELKGIDFKE	3.72
DTLVNRIELKGIDFKEDGNILGHKLEYNYNSHNVYIMA	1.23

[a]The cross-correlation score between acquired and theoretical spectra

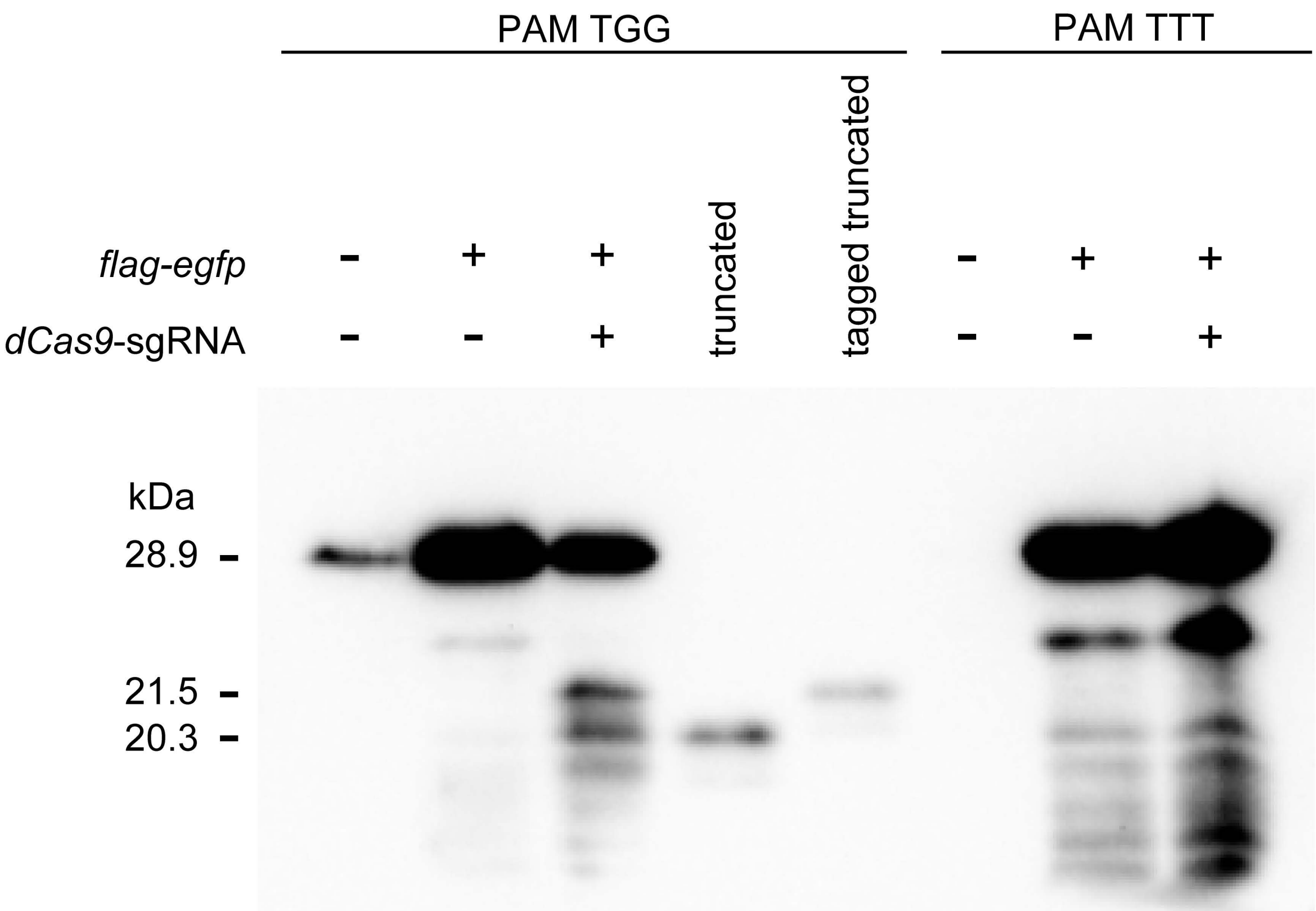

Fig. S1. dCas9-sgRNA truncation and tagging by tmRNA-SmpB requires a functional PAM site. FLAG-EGFP with either the native PAM site (TGG) or the PAM site mutated to TTT was expressed with dCas9-sgRNA in *E. coli* SsrA-H6D and detected with α-FLAG antibody. Strains expressing truncated FLAG-EGFP (residues 1-179) and truncated FLAG-EGFP with the SsrA-H6D tag at its C-terminus are shown as size markers.

B	B Ions	B+2H	B-NH3	B-H2O	AA	Y Ions	Y+2H	Y-NH3	Y-H2O	Y
1	116.0	58.5		98.0	D	2,181.2	1,091.1	2,164.2	2,163.2	18
2	244.1	122.6	227.1	226.1	K	2,066.2	1,033.6	2,049.2	2,048.2	17
3	372.2	186.6	355.2	354.2	Q	1,938.1	969.6	1,921.1	1,920.1	16
4	500.3	250.6	483.3	482.3	K	1,810.0	905.5	1,793.0	1,792.0	15
5	614.3	307.7	597.3	596.3	N	1,681.9	841.5	1,664.9	1,663.9	14
6	671.3	336.2	654.3	653.3	G	1,567.9	784.5	1,550.9	1,549.9	13
7	784.4	392.7	767.4	766.4	I	1,510.9	755.9	1,493.9	1,492.9	12
8	912.5	456.8	895.5	894.5	K	1,397.8	699.4	1,380.8	1,379.8	11
9	1,011.6	506.3	994.6	993.6	V	1,269.7	635.4	1,252.7	1,251.7	10
10	1,125.6	563.3	1,108.6	1,107.6	N	1,170.6	585.8	1,153.6	1,152.6	9
11	1,272.7	636.9	1,255.7	1,254.7	F	1,056.6	528.8	1,039.6	1,038.6	8
12	1,400.8	700.9	1,383.8	1,382.8	K	909.5	455.3	892.5	891.5	7
13	1,513.9	757.4	1,496.9	1,495.9	I	781.4	391.2	764.4	763.4	6
14	1,670.0	835.5	1,653.0	1,652.0	R	668.3	334.7	651.3	650.3	5
15	1,807.0	904.0	1,790.0	1,789.0	H	512.2	256.6	495.2	494.2	4
16	1,921.1	961.0	1,904.1	1,903.1	N	375.2	188.1	358.2	357.2	3
17	2,034.2	1,017.6	2,017.1	2,016.2	I	261.1	131.1		243.1	2
18	2,181.2	1,091.1	2,164.2	2,163.2	E	148.1	74.5		130.0	1

Fig. S2. Fragment ions of FLAG-EGFP. The predicted fragment ions for the peptide sequence DKQKNGIKVNFKIRHNIE (diagrammed in Figure 2C) are shown. Colored boxes indicate ions that were recovered in the MS/MS spectrum.

B	B Ions	B+2H	B-NH3	B-H2O	AA	Y Ions	Y+2H	Y-NH3	Y-H2O	Y
1	116.0	58.5		98.0	D	2,111.0	1,056.0	2,093.9	2,093.0	18
2	244.1	122.6	227.1	226.1	K	1,995.9	998.5	1,978.9	1,977.9	17
3	372.2	186.6	355.2	354.2	Q	1,867.8	934.4	1,850.8	1,849.8	16
4	500.3	250.6	483.3	482.3	K	1,739.8	870.4	1,722.8	1,721.8	15
5	614.3	307.7	597.3	596.3	N	1,611.7	806.3	1,594.7	1,593.7	14
6	671.3	336.2	654.3	653.3	G	1,497.6	749.3	1,480.6	1,479.6	13
7	784.4	392.7	767.4	766.4	I	1,440.6	720.8	1,423.6	1,422.6	12
8	855.5	428.2	838.4	837.5	A	1,327.5	664.3	1,310.5	1,309.5	11
9	926.5	463.8	909.5	908.5	A	1,256.5	628.8	1,239.5	1,238.5	10
10	1,040.5	520.8	1,023.5	1,022.5	N	1,185.5	593.2	1,168.4	1,167.5	9
11	1,155.6	578.3	1,138.5	1,137.6	D	1,071.4	536.2		1,053.4	8
12	1,292.6	646.8	1,275.6	1,274.6	H	956.4	478.7		938.4	7
13	1,429.7	715.4	1,412.7	1,411.7	H	819.3	410.2		801.3	6
14	1,566.8	783.9	1,549.7	1,548.7	H	682.3	341.6		664.3	5
15	1,703.8	852.4	1,686.8	1,685.8	H	545.2	273.1		527.2	4
16	1,840.9	920.9	1,823.8	1,822.9	H	408.2	204.6		390.2	3
17	1,977.9	989.5	1,960.9	1,959.9	H	271.1	136.1		253.1	2
18	2,111.0	1,056.0	2,093.9	2,093.0	D	134.0	67.5		116.0	1

Fig. S3. Fragment ions of tagged FLAG-EGFP. The predicted fragment ions for the tagged peptide sequence DKQKNGIAANDHHHHHHD (diagrammed in Figure 2C) are shown. Colored boxes indicate ions that were recovered in the MS/MS spectrum.

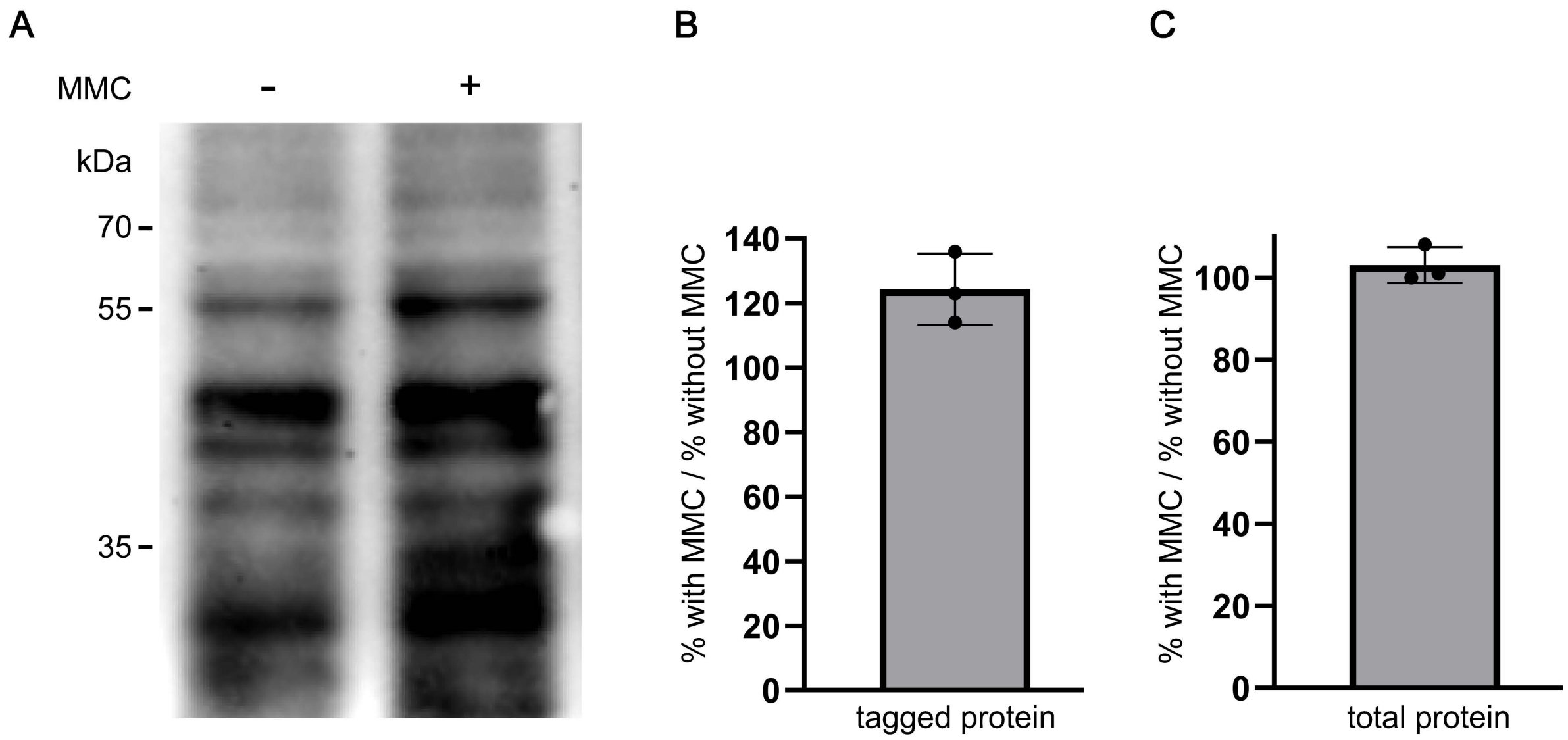

Fig. S4. SsrA-H6D tagging in cells treated with MMC. (*A*) *E. coli* containing the SsrA-H6D tag was treated with MMC and the percent tagging relative to untreated was determined by western blot with Penta-His antibody. Tagging in the untreated sample was defined as 100%. (*B*) Bar plot showing the percent tagging in treated cells relative to untreated. (*C*) Bar plot showing the total protein determined by densitometry of Ponceau stained blots with the untreated sample set to 100%. Data are shown as the mean ± standard deviation of three biological replicates.

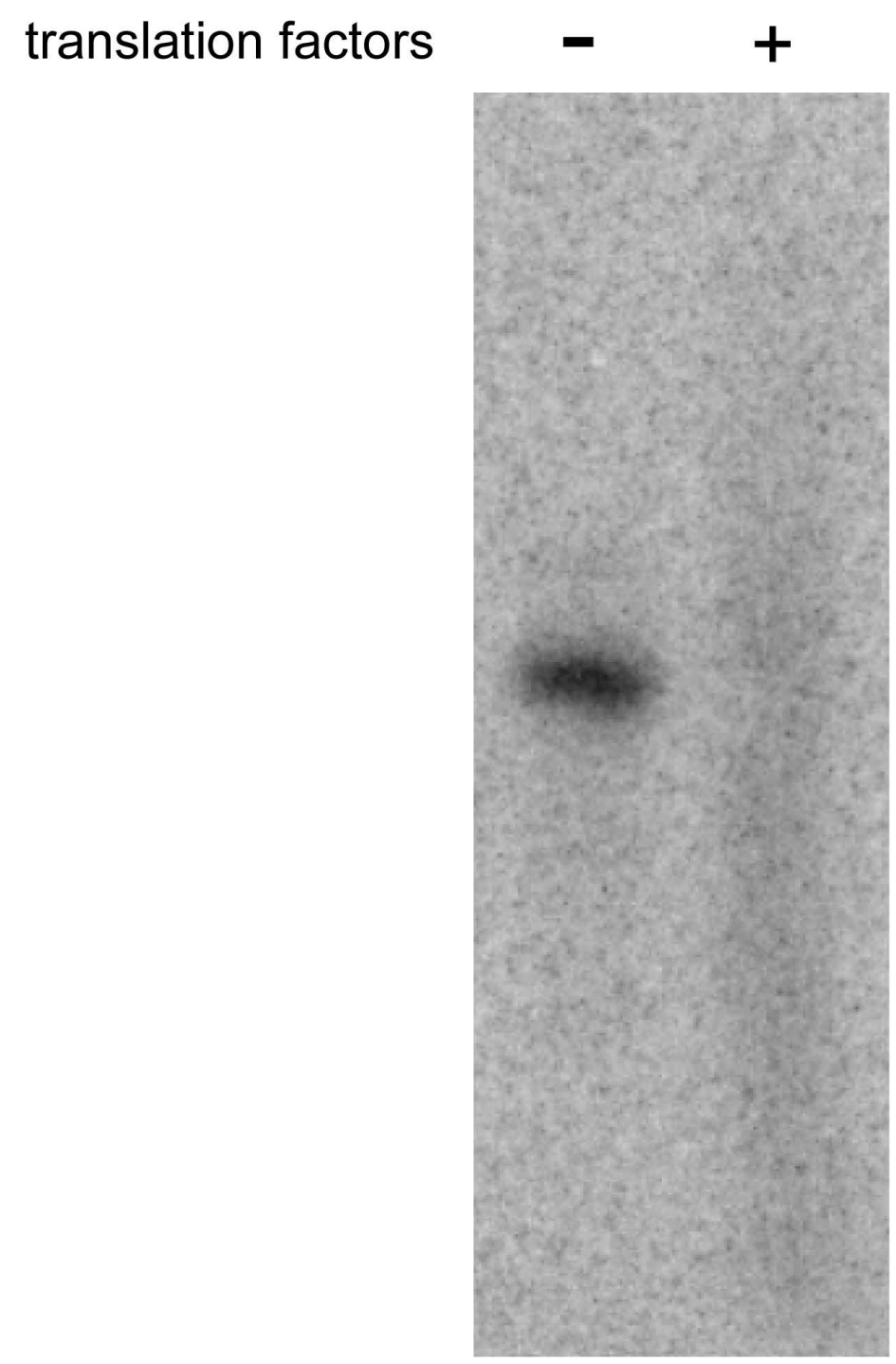

Fig. S5. Released transcript is rapidly degraded *in vitro*. Biotinylated DHFR template DNA without dCas9-sgRNA was transcribed *in vitro* and then incubated with streptavidin beads. The supernatant containing released transcript was split into equal volumes. Translation factors and ribosomes were added to one of the split reactions and an equal volume of transcription buffer was added to the other reaction. Both reactions were incubated at 37C for 10 min, electrophoresed, and phosphorimaged.

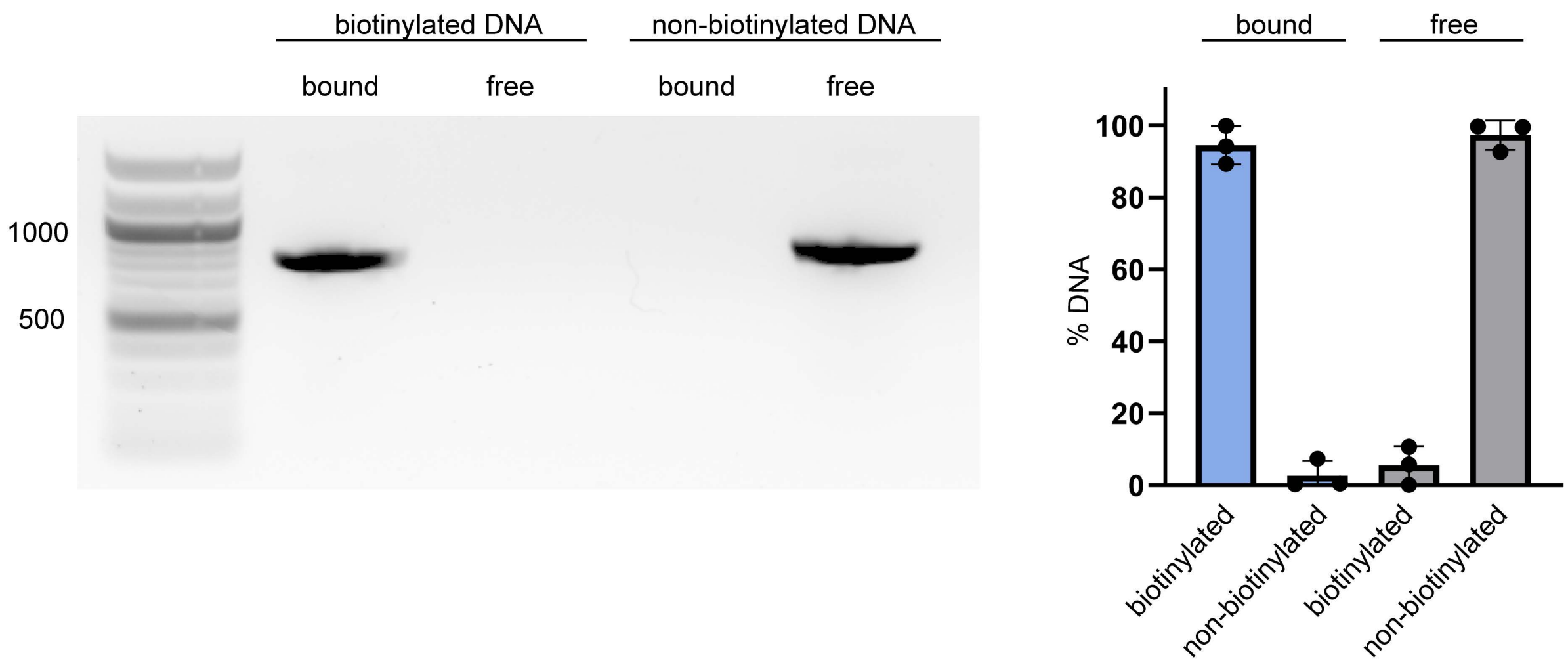

Fig. S6. Streptavidin association is specific for biotinylated DNA. Biotinylated or non-biotinylated DHFR DNA template was bound to streptavidin beads, followed by separation of the bound and free fractions. The DNA was electrophoresed and the percent of DNA in each fraction was quantified by densitometry.

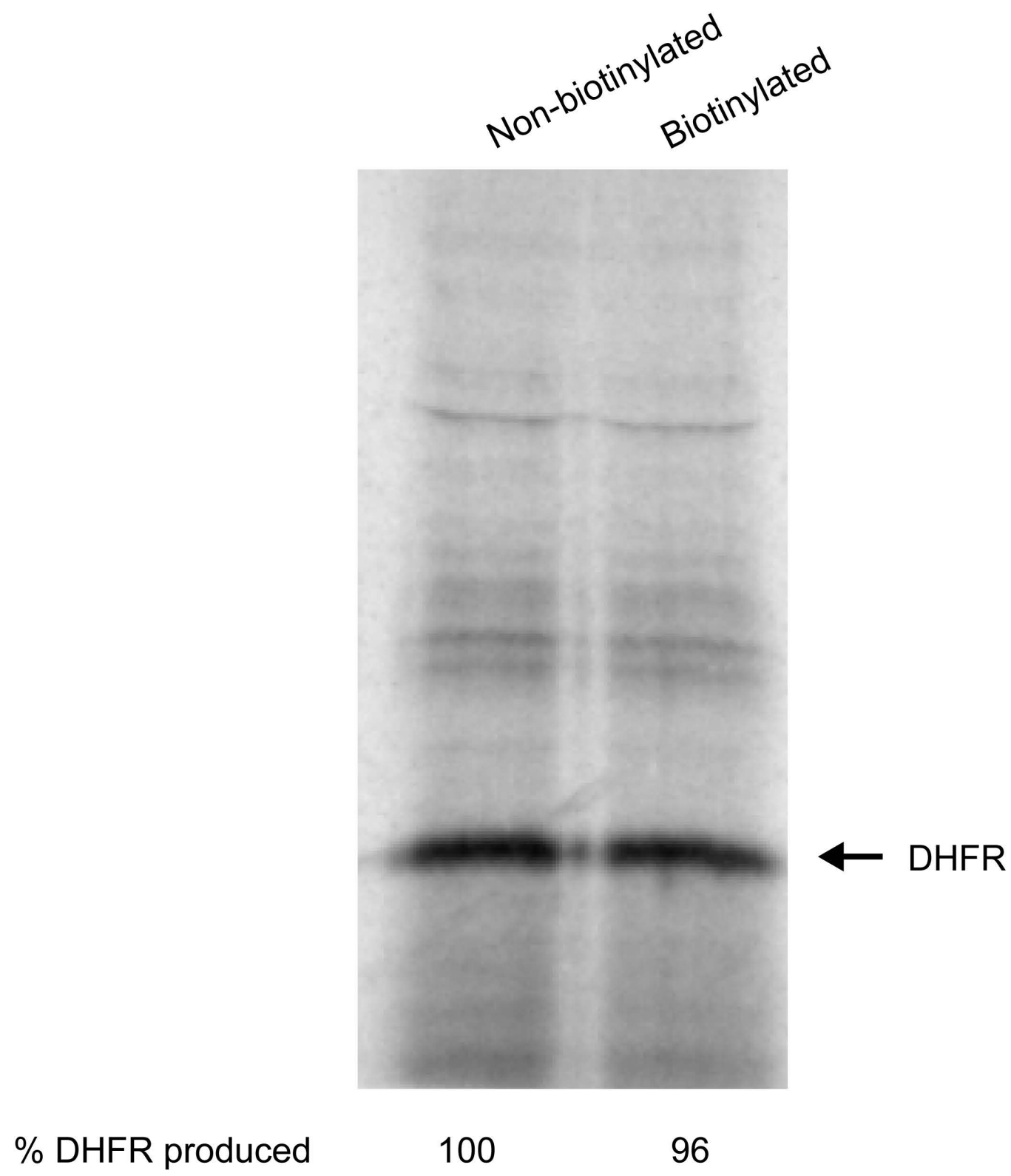

Fig. S7. Biotinylation of DHFR template DNA does not affect translation yield. DHFR template DNA with or without 5’ biotinylation was used as template for *in vitro* transcription and translation as described in the methods. Translation was monitored by incorporation of [^{35}S]-methionine and detected by phosphorimaging. The amount of DHFR protein produced from biotinylated DHFR template was calculated as a percentage relative to non-biotinylated template DNA.

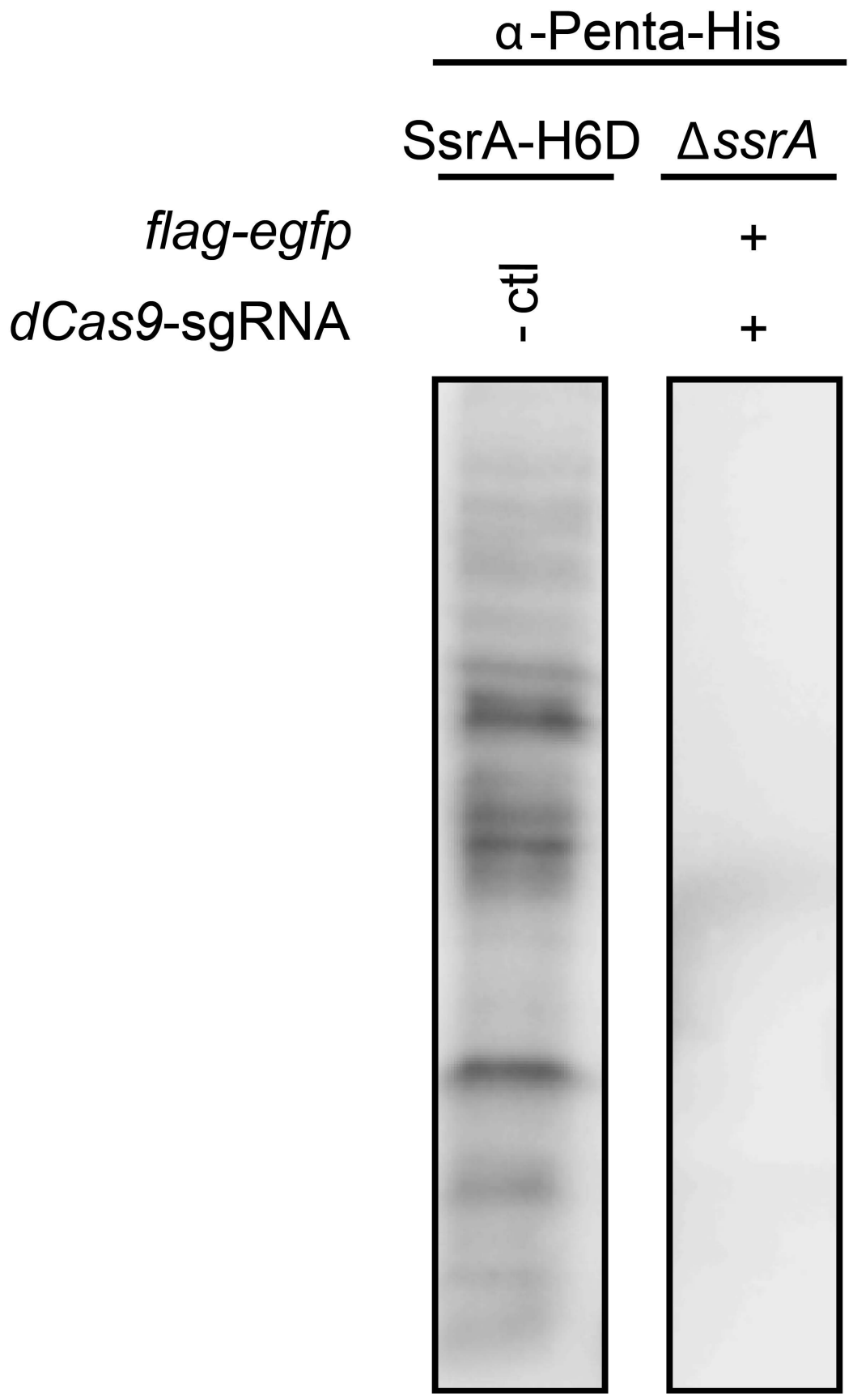

Fig. S8. α-Penta-His antibody is specific to his-tagged proteins. His-tagged proteins or proteins that cross-reacted with the α-Penta-His antibody were analyzed by western blotting in *E. coli* strains containing a variant tmRNA with the SsrA-H6D tag (ANDHHHHHHD) or with no tmRNA (Δ*ssrA*). Tables above the blots indicate induction of FLAG-EGFP and dCas9-sgRNA. A strain with no plasmids is included as a negative control (-ctl) for background SsrAH6D-tagging that occurs independently of FLAG-EGFP and dCas9-sgRNA expression. The two controls are from the same blot, with unnecessary lanes removed.
